# Sterilization in Dental Practice

**Published:** 1899-10

**Authors:** T. C. Van Kirk

**Affiliations:** Alleghany, Pa.


ARTICLE II.

### STERILIZATION IN DENTAL PRACTICE.

T. C. VAN KIRK, D. D. S., ALLEGHANY, PA.

**(A paper read before the Odontological Society of Western Pennsylvania, at Pittsburg, March 14, 1899.)**

ABSTRACT.

Sterilization may be defined as the process of destroying all spores and germs, with the view to prevent the development of bacterial or other organisms.

To the surgeon it is not only important to destroy all germs which may have found lodgment on the instruments and appliances employed in his operations, in order to prevent the infection of his patient by pathogenic organisms, but also to be scrupulously careful after the operation to prevent their admission or inhibit their growth by the use of antiseptic dressings : in other words, complete sterilization is the "Sine qua non" of the careful surgeon of the present day.

The importance of, or necessity for sterilization, implies the probable presence of pathogenic bacteria in all cases operated upon. Miller has demonstrated that there are twenty-two mouth bacteria; of these, eight or ten are almost constantly met with, and six are invariably present. Besides these mouth bacteria, nearly all of which are non-pathogenic, almost every minute organism which has been described as growing in any position, has been found in the mouth. In other words, the mouth is the typical incubating chamber for the culture of almost all germs. During epidemics or when persons come in contact with those suffering from various zymotic diseases the organisms associated with them are frequently taken into the mouth.

Surgeon-General Sternberg, of the United States army, an eminent authority on bacteriology, states that he has found the pneumo-coccus constantly present in his own mouth, and that he has repeatedly caused pneumonia, followed by death, in rabbits, by injecting them with the saliva taken from his mouth when in a state of perfect health.

Numerous authorities might be quoted substantiating the foregoing statements, but time will not permit. I have thus shown that even in the most healthy mouths, to say nothing of these contaminated by disease; such as tuberculosis, erysipelas, syphilis, etc., there may be present micro-organisms, which, becoming attached to our instruments, may by accident or otherwise be introduced into the system of the patient himself or of some succeeding patient, or even of the operator, with calamitous results.

Muir and Ritchie in their Manual of Bacteriology say that all bacteria can be killed either by heat, drying, starvation, or by chemical agents; of these, chemical agents commonly called germicides, are most frequently used. As a general rule the two chemical agents which heretofore have most frequently been used are a 1 to 20 solution of carbolic acid, and a 1 to 1,000 solution of bichloride of mercury. But these do not meet all the requirements of a sterilizing agent for a dental office, not only because of the time required in some cases for complete sterilization, but because of the corrosive action on steel instruments, and because some articles would be destroyed by being immersed in these solutions. Many have, therefore, taken advantage of the principal that all bacteria are destroyed by heat, and have advocated this method of sterilization. This, again, presents disadvantages to the dentist, as the high temperature necessary to kill the spores of some bacilli, injures the temper of delicate steel instruments.

The hands of the operator should be washed and disinfected after each operation; not as an act of cleanliness merely, but as a safeguard, for himself and succeeding patient, against possible infection from a previous patient.

My own method after washing, is to apply to the hands a small quantity of borolyptol, allowing it to dry upon the skin.

The mouth of the patient may be rendered practically sterilized by use of some one of the many good antiseptics that are now available. Of these, borolyptol is coming into popular favor. A small quantity held in the mouth for a few minutes arrests the growth of the microorganisms. Tubes of agar planted with saliva from the mouth, after exposure to borolyptol, shows no growth after forty-eight hours incubation, while tubes inoculated from the same mouth before the application just stated gave abundant growth, thus showing that borolyptol has at least the power of inhibiting the growth of mouth bacteria.

With regard to borolyptol, I think it might be safely used for sterilizing instruments, as only a slight tarnish was formed on steel instruments immersed in it for thirty-six hours, but this is longer than is necessary to accomplish sterilization; half an hour's exposure produced no tarnish whatever.—*Dental Brief*,

